# Phase-Resolved Two-Dimensional Infrared Spectroscopy of Solution-Phase Vibrational Polaritons on Gold Antenna Meta-Surfaces

**DOI:** 10.1021/acs.jpclett.6c00009

**Published:** 2026-02-22

**Authors:** Shmuel Sufrin, Bar Cohn, Lev Chuntonov

**Affiliations:** Schulich Faculty of Chemistry, 26747 Technion - Israel Institute of Technology, Haifa 3200003, Israel; Solid State Institute, Technion - Israel Institute of Technology, Haifa 3200003, Israel; The Helen Diller Quantum Center, Technion - Israel Institute of Technology, Haifa 3200003, Israel

## Abstract

Vibrational polaritons are quasiparticles that form when optically allowed molecular vibrations strongly couple to photonic resonances of infrared cavity. Understanding vibro-polariton properties is essential to harness their potential in fields from synthetic chemistry to quantum technologies. We studied vibro-polaritons generated with infrared meta-surfaces—high-optical-quality arrays of gold microantennas. Linear and third-order nonlinear two-dimensional infrared spectroscopy (2DIR) combined with electromagnetic analysis emphasized, on one hand, the surface-confined character of vibro-polariton waves and their quantum state nature on the other. Phase-resolved 2DIR line shapes highlighted the anharmonic character of vibro-polaritons and revealed coupling with the excitation of molecular-like reservoir states. Experimental data were qualitatively described by nonlinear response functions with signal phases obtained from electromagnetic calculations and anharmonic constants of few wavenumbers. Such agreement allows us to rule out alternative scenarios like selective signal enhancement from inhomogeneous distribution of molecular frequencies or signal amplification by weak coupling to photonic resonances.

## Graphical abstract

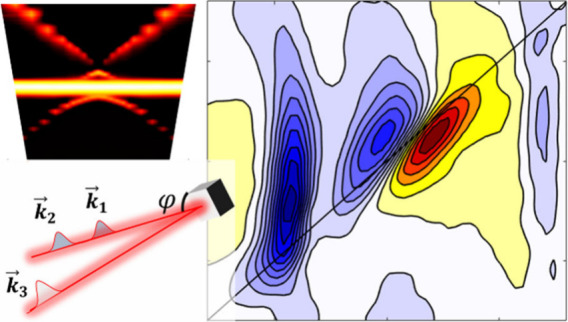

Vibrational polaritons are formed when optically allowed molecular vibrational transitions strongly interact with a photonic mode of infrared optical cavity,^1–3^ which can be constructed with resonators of various designs.^4–8^ Because the transition dipoles of molecular vibrations are relatively weak (a few tenths of a Debye), numerous molecules (*N* ∼ 10^9^–10^10^) are required to observe distinct vibro-polariton transitions. Polaritons are viewed as linear combinations of the collective bright state, where transitions of all *N* participating molecules contribute in-phase,^9^ and a photonic mode; therefore, they share the properties of both molecular and photonic components.^10^ A great interest in molecular polaritons emerged following the proposal of a novel paradigm,^11^ where various chemical processes, reaction mechanisms included, can be controlled via polariton states even without their direct excitation by light; instead, these processes are activated by fluctuating fields of an electromagnetic vacuum.^12–14^ Despite over a decade of intensive research, the field of polariton chemistry remains controversial, with a continuous flux of experimental and theoretical reports both supporting and rejecting the paradigm of vacuum field chemistry.^15–21^ Conventional explanations, i.e., not involving vacuum fields, were found sufficient to describe certain experiments involving photonic resonators.^22–25^ Theoretical progress in the field meets challenges because of the enormous amount of degrees of freedom in a system, where the collective reactivity of *N* polyatomic molecules needs to be considered.^9,26–29^

In addition to polaritons, *N*-1 reservoir states are also formed in the strongly coupled system; their properties are considered to be largely molecular. Since the density of reservoir states greatly outnumbers that of polaritons (*N*-1 vs 2), any process involving the polariton density of states appears to be noncompetitive.^30^ However, a certain degree of reservoir delocalization was predicted,^31,32^ potentially leading to cooperative many-body behavior.^33–35^ When polaritons are formed by molecular excitation interacting with the optical mode of a Fabry–Perot cavity,^1–3^ only polariton transitions appear in a transmission spectrum, whereas the reservoir states cannot be easily observed.^36,37^ When polaritons are formed by interacting with a photonic mode of an open cavity, e.g., a meta-surface of a periodic array of dipole antennas, which supports surface-lattice resonances (SLRs),^4,8,21^ reservoir states readily appear in the transmission spectrum and their properties can be investigated spectroscopically, once the problem of the background signal from the uncoupled molecules that are always present in the sample is efficiently solved.

Despite their low density of states, polaritons are intriguing quasiparticles with unique properties of both fundamental scientific and applicative technological interest.^38,39^ Transient absorption and two-dimensional infrared (2DIR) spectroscopies were employed in recent studies of various molecular vibrationally strongly coupled systems.^25,40,41^ Nonlinear spectroscopy of vibro-polaritons reveals their ultrafast dynamics, the details of their interaction with the environment, and even changes that mixing with a photonic mode can impose on the molecular chemical transformations.^36,42–44^ Observation of nonlinear spectroscopic signals requires anharmonic potentials.^45^ Despite the anharmonic character of the molecular vibrational component, vibro-polaritons involving a large number of molecules are predicted to be largely harmonic, which implies that the corresponding nonlinear signals vanish.^46–48^ To date, experimental measurements could not quantitatively determine these very small anharmonic constants and only their upper bounds were estimated.^49^ To interpret the experimental nonlinear spectroscopic signals, a model was proposed that focuses on the first excitation manifold of the strongly coupled system, assuming that the coupling strength is reduced with the fraction of the excited molecules, which leads to contraction of the polaritonic Rabi splitting.^50,51^ More advanced models explicitly considered the higher excited states manifold of the strongly coupled system;^47,52^ however, direct experimental observations of these states in vibro-polaritons are yet to appear.

In the regime of vanishing nonlinearity, for the strongly coupled systems based on Fabry–Perot cavities, it was proposed that optical transmission at polariton frequencies can serve as an effective optical filter, which selects nonlinear signals from the inhomogeneously distributed bare-molecule transition frequencies.^25,53^ Regarding open cavity meta-surfaces involving metallic antennas,^4,54^ enhanced near-fields induced at the polariton transition frequencies can potentially play a similar role of a selective filter. Recently, however, several indirect observations were made that argue against this scenario. First, the strength of the polariton and bare-molecule transitions was shown to have a different excitation power dependence:^41^ polariton signals saturated at a relatively low excitation laser power, as expected for a system with a finite number of emitters,^55^ whereas a bare-molecule signal did not saturate even at the maximal excitation laser power available in the experimental setup, as expected for a system with a very large number of emitters available for excitation. In a different study, the ultrafast dynamics of nonlinear signals involving vibro-polaritons significantly differed from that of the bare-molecule signals enhanced by the near-fields of the SLR modes.^56^ Since polaritons are only partially molecular, their intramolecular relaxation rates were slowed down twice, as opposed to the enhanced molecular signals, whose relaxation dynamics were identical to the bare-molecule case.

In the present work, we performed measurements of the third-order nonlinear 2DIR line shapes of molecular polaritons formed by the interaction between the C–N stretching vibrational mode of ammonium thiocyanate and the SLR modes of an infrared half-wavelength antenna array meta-surface. 2DIR facilitates the analysis of spectrally congested data by spreading it in two dimensions and reveals the ultrafast dynamics of correlation between different excitations, which reflects the changes the system undergoes on these time scales.^48^ Therefore, there is a strong motivation to employ 2DIR spectroscopy in studies of vibrationally strongly coupled systems. 2DIR spectra are typically phase resolved, such that both purely absorptive and dispersive components of the complex line shape can be measured.^48,57,58^ Usually, it is the absorptive part that is of interest, since it provides narrow intuitively interpretable line shapes; therefore, it is the one that is typically reported.^59^ However, phase shifts experienced by the signal light at metallic surfaces mix between the absorptive and dispersive line shape components.^60–63^

In the present work, phase-resolved 2DIR spectra of vibro-polaritons enabled us to examine the possibility that the nonlinear signals observed at the polariton transition frequencies are, in fact, bare-molecule signals, which were selectively amplified from the inhomogeneously broadened ensemble by the enhanced near fields around metallic antennas. Interestingly, merely applying a physically sound narrowband amplification filter function to the bare-molecule data failed to reproduce the experimental spectra of the strongly coupled system. Modeling of a 2DIR spectrum under the assumptions of weak coupling between the inhomogeneously broadened molecular transitions and polaritons also did not agree with the experimental results. On the other hand, modeling of a 2DIR spectrum based on third-order nonlinear response functions,^64^ where small anharmonicities and phase shifts of the polariton transitions with respect to the bare-molecule were assumed, qualitatively reproduced all the features of the experimental spectra.

Surprisingly, we found that polariton transitions with strong extinction in linear transmission do not necessarily show strong peaks in 2DIR. Indeed, extinction measured in the linear spectrum represents not only absorption, but also scattering, reflection, and diffraction. It is well-known in surface-enhanced Raman spectroscopy that both large absorption and scattering cross sections are generally needed for efficient signal enhancement.^65^ Similar considerations also apply for surface-enhanced 2DIR.^66–69^ However, SLR-based vibro-polaritons are more complex. Since the SLRs are surface-confined waves, molecular polaritons associated with them are also confined to the surface and their in-plane diffraction is observed in the linear extinction spectrum. The nonlinear 2DIR signals are stimulated by the excitation laser light and obey momentum conservation. Thus, any polariton signal emitted via the four-wave mixing process into the phase-matching direction involves four in-plane diffraction events. By the principle of reciprocity, efficient coupling of the incident light to the in-plane modes via diffraction correlates with efficient out-coupling of the stimulated nonlinear signal;^70^ however, our experimental results indicate that there is no simple correlation between the strength of the linear and nonlinear polariton signals. We found that, in addition to the in-plane diffraction and absorption, out-of-plane diffraction contributes to linear polariton extinction, whereas for the nonlinear signal strength, anharmonicity is known to play a significant role. All these factors need to be considered to understand the phase-resolved 2DIR signals in detail.

This manuscript is organized as follows: First, with the help of simple theoretical models, we describe how surface-confined vibro-polaritons form and how we use these models to interpret linear spectroscopic measurements. Next, we present the results of phase-resolved 2DIR spectroscopy, which are the main subject of the present work, and discuss their interpretation. Specifically, the surface-enhancement and weak coupling of the bare-molecule signals hypotheses are ruled out because they are not supported by experimental data. Finally, we demonstrate that 2DIR results can be qualitatively described by the formalism of the third-order nonlinear response functions,^64^ supported by electromagnetic numerical simulations.

## Vibro-Polaritons Formed with SLRs Are Surface-Confined Waves

Surface-lattice resonances in periodic arrays of dipolar scatterers are hybridized modes formed by the interaction between the broadband resonance of the site scatterer and the diffraction order of the point-like lattice,^69^ which is essentially infinitely narrowband. To achieve strong vibrational coupling, a high-quality SLR is required (Q≳100); therefore, hybridized modes with a large contribution of the diffraction order are typically chosen. Thus, the characteristics of the broadband site can be neglected to the lowest order approximation and the SLR state, which is within such approximation frequently referred to as Rayleigh anomaly, is associated with the vanishing out-of-plane component of the wavevector,

1 $$k_{⊥}=\sqrt{{\left|{\mathrm{k⃗}}_{i}\right|}^{2}-{\left|{\mathrm{k⃗}}_{∥}\right|}^{2}}$$

where ${\mathrm{k⃗}}_{i}$ is the incident wavevector with total momentum $\left|{\mathrm{k⃗}}_{i}\right|=\frac{n\omega }{c}$ , *n* is the refractive index of the surrounding medium, *c* is the speed of light, ${\mathrm{k⃗}}_{∥}=$ ${\mathrm{k⃗}}_{i,∥}$ $+{\mathrm{k⃗}}_{G}$ is the in-plane wavevector, ${\mathrm{k⃗}}_{i,∥}$ is the in-plane component of ${\mathrm{k⃗}}_{i}$ , ${\mathrm{k⃗}}_{G}=$ $\frac{2\pi }{\Lambda_{x}}l\mathrm{x̂}+$ $\frac{2\pi }{\Lambda_{y}}mŷ$ is the lattice wavevector, Λ_*x*_ and Λ_*y*_ are lattice constants, and *l* and *m* are diffraction orders. When *k*_⊥_ = 0, the corresponding electromagnetic wave is confined to the surface.

The phenomenon described by eq 1 is illustrated in Figure 1. The scanning electron microimage of an array of half-wavelength gold antennas is shown in panel a. The array has lattice constants Λ_*x*_ = 3.4 μm, Λ_*y*_ = 1.2 μm; the antenna length is *L* = 0.75 μm, which corresponds to the site resonance wavelength of λ_*a*_ = 2.8 μm (ω_*a*_= 3,600 cm^–1^) given by the relation λ_*a*_ = 2*Ln* + *c*, where *n* = 1.44 is the refractive index of the *N’,N’*-dimethylformamide solvent (DMF) used in the experiments in the spectral region of interest away from its vibrational resonances,^71^ and *c* is the constant related to the shape of the antenna tips.^72^ As seen in Figure 1b, a collective SLR mode emerges at λ_SLR_ = 4.9 μm (ω_SLR_ = 2050 cm^–1^), such that ω_SLR_ is detuned sufficiently far to the red from ω_*a*_ and the contribution of the broadband site resonance can indeed be neglected. The frequency dependence of *k*_⊥_, obtained with eq 1 for the diffraction order (+1,0), is plotted in Figure 1b: *k*_⊥_ is purely imaginary for frequencies below ω_SLR_ and purely real for frequencies above ω_SLR_; the magnitude |*k*_⊥_| vanishes at the resonance, indicating that SLR is a surface-confined wave.

**Figure 1:**
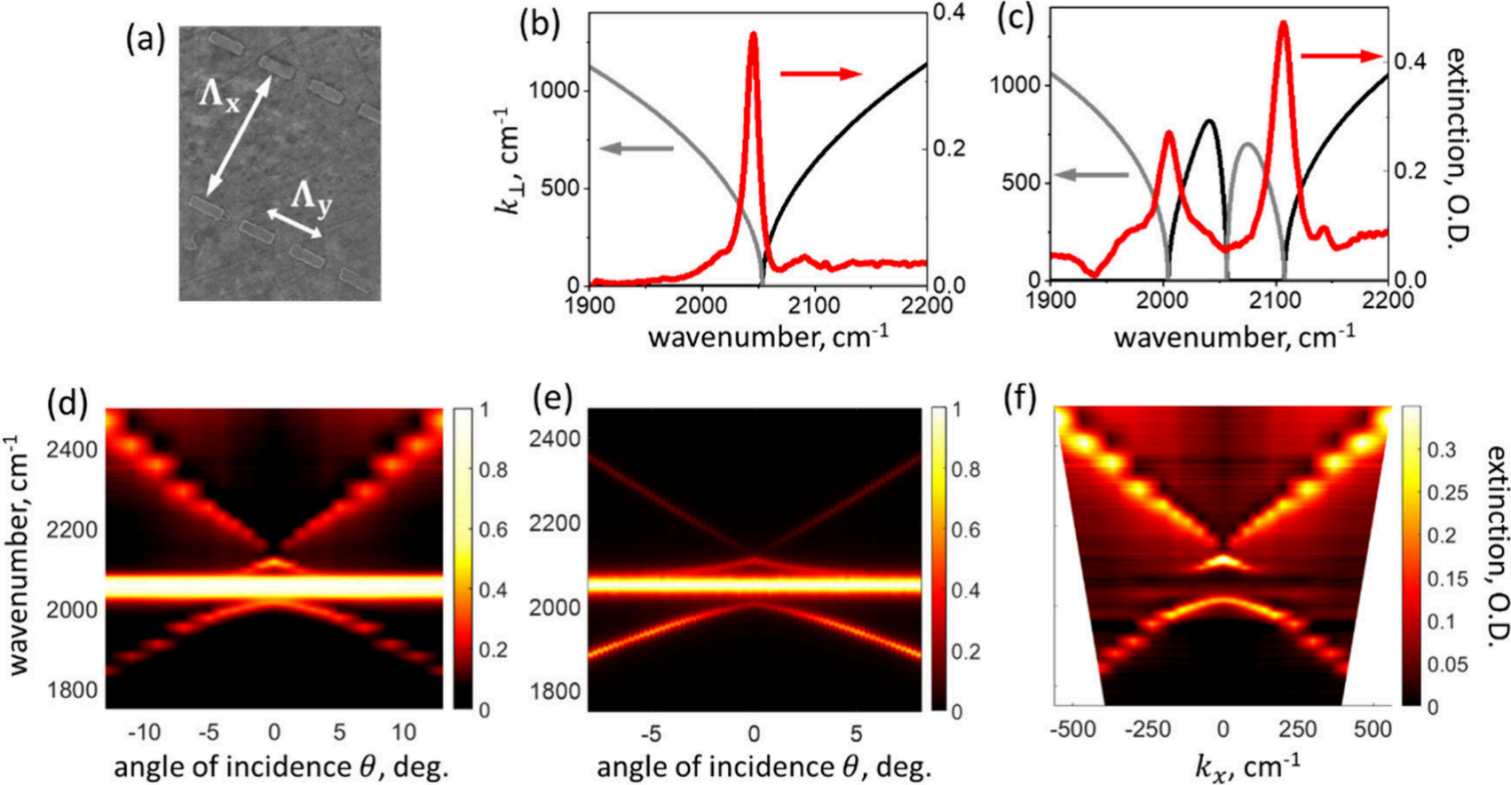
**Linear spectroscopy of SLR and vibro-polaritons.** (a) Scanning electron micrograph of the antenna array; the lattice constants are Λ_*x*_ = 3.4 μm and Λ_*y*_ = 1.2 μm. (b) Left abscissa – the out-of-plane component *k*_⊥_ of the diffracted wave vector. Black line – the real part, the gray line – the imaginary part. Right abscissa – the spectrum of the antenna array supporting SLR covered by 500 nm-thick DMF film and measured at normal incidence and TE (ŷ) light polarization. (c) Same as in panel b, but for the film of a 2 M ammonium thiocyanate solution in DMF. The bare-molecule solution reference spectrum was subtracted. (d) Dispersion map of the strongly coupled sample: the extinction spectra measured for different angles of incidence of TE-polarized light. (e) Spectrum calculated based on the Tavis-Cummings-like model including inhomogeneous broadening of molecular transitions (see the text for details). (f) Same as in panel d, but with the bare-molecule spectrum subtracted and the angle of incidence converted to the in-plane momentum.

Next, we use eq 1 to evaluate *k*_⊥_ for the lattice covered with 500 nm-thick film of 2 M ammonium thiocyanate solution in DMF, where C–N stretching vibrational transition appears at ω_CN_ = 2050 cm^–1^. The frequency dependence of the solution refractive index was obtained from its linear infrared absorption spectrum using the Kramers–Kronig relations (Hilbert transform); the resulting values of *k*_⊥_ are shown in Figure 1c. We found that *k*_⊥_ changes from purely imaginary to purely real not only at ω_SLR_, but also at two new frequencies, which match the frequencies of the upper (UP) and lower (LP) polariton transitions in the linear spectrum of the antenna array covered with the thiocyanate solution film shown in Figure 1d. It is clear that *k*_⊥_ vanishes for polaritons, which are, therefore, surface-confined waves.

Strongly coupled systems are frequently analyzed within the framework of the Tavis-Cummings (TC) model, where *N* emitters are coupled to a single photonic cavity mode. In Figure 1d the dispersion relations obtained from the linear extinction measurements of our sample are shown for the TE polarization of the incident light. These data are closely reproduced by the TC-like model, which accounts for two SLR modes^4,73^ (+1,0) and (−1,0). The coupling strength between the molecules and SLR modes used in the model was obtained from the corresponding local electric fields evaluated with electromagnetic numerical simulations (see Supporting Information). In addition, our model accounts for inhomogeneous distribution of molecular transition frequencies obtained from the analysis of the corresponding 2DIR line shape^48^ (see below) and the experimental oscillator strength of the molecular transition, which is similar to that of the SLR. Detailed mathematical description of the model is given in section S1 of Supporting Information. The results of the model shown in Figure 1f are fully consistent not only with linear spectroscopy in panel d, but also with 2DIR data discussed below. The good agreement with the spectroscopic results validates its use in estimating the molecular and SLR components’ contribution to the vibro-polariton states; this is quantified by Hopfield coefficients *c*_*SLR*(*m*)_^*UP*(*LP*)^, where ${\left|c_{+1,0}^{UP\left(LP\right)}\right|}^{2}+$ ${\left|c_{-1,0}^{UP\left(LP\right)}\right|}^{2}+$ ${\left|c_{m}^{UP\left(LP\right)}\right|}^{2}=1$ .

## 2DIR Spectroscopy of Vibro-Polaritons

In cases of isolated homogeneous transitions, proper phasing of 2DIR signals allows one to separate the absorptive and dispersive components of the complex-valued line shapes. The former is given by the real part of the 2D spectrum, which includes the sum of the signals from both the rephasing and nonrephasing excitation pathways,^59^ whereas the latter can be obtained from their difference.^48,57^ The imaginary part of the 2D data is interpreted as a transient dispersive part of the line shape.^58^

The 2DIR spectra of the CN stretching vibration of 2 M solution of ammonium thiocyanate in DMF collected at the waiting time *T*_w_ = 300 fs are shown in Figure 2 (top row, panels a2-a4). A choice of such a high molecular concentration was dictated by the Rabi frequency scaling, $\Omega \propto \sqrt{N}$ , where *N* is the number of molecules occupying the volume of the SLR mode. High concentration leads to broadening of the linear molecular line shapes compared to low concentration samples (Δω = 40 cm^–1^ vs 15 cm^–1^, fwhm) as seen in Figure 2a1, whereas 2DIR line shapes are elongated along the diagonal. The broadening reflects different types of microscopic intermolecular interactions associated with high concentration samples, including ion pairing, aggregation, etc., which under the conditions of molecular disorder are all averaged out and result in a continuous Gaussian line shape. Since in the present case no specific ion pair or exciton peaks can be resolved, we phenomenologically interpreted the broad line shape as inhomogeneous distribution of the molecular transition frequencies. Large inhomogeneity leads to an imbalance between the rephasing and nonrephasing signal amplitudes such that the dispersive part of the line shape is not fully eliminated by taking the real part of the 2D spectrum and, consequently, the corresponding peaks appear tilted.

**Figure 2:**
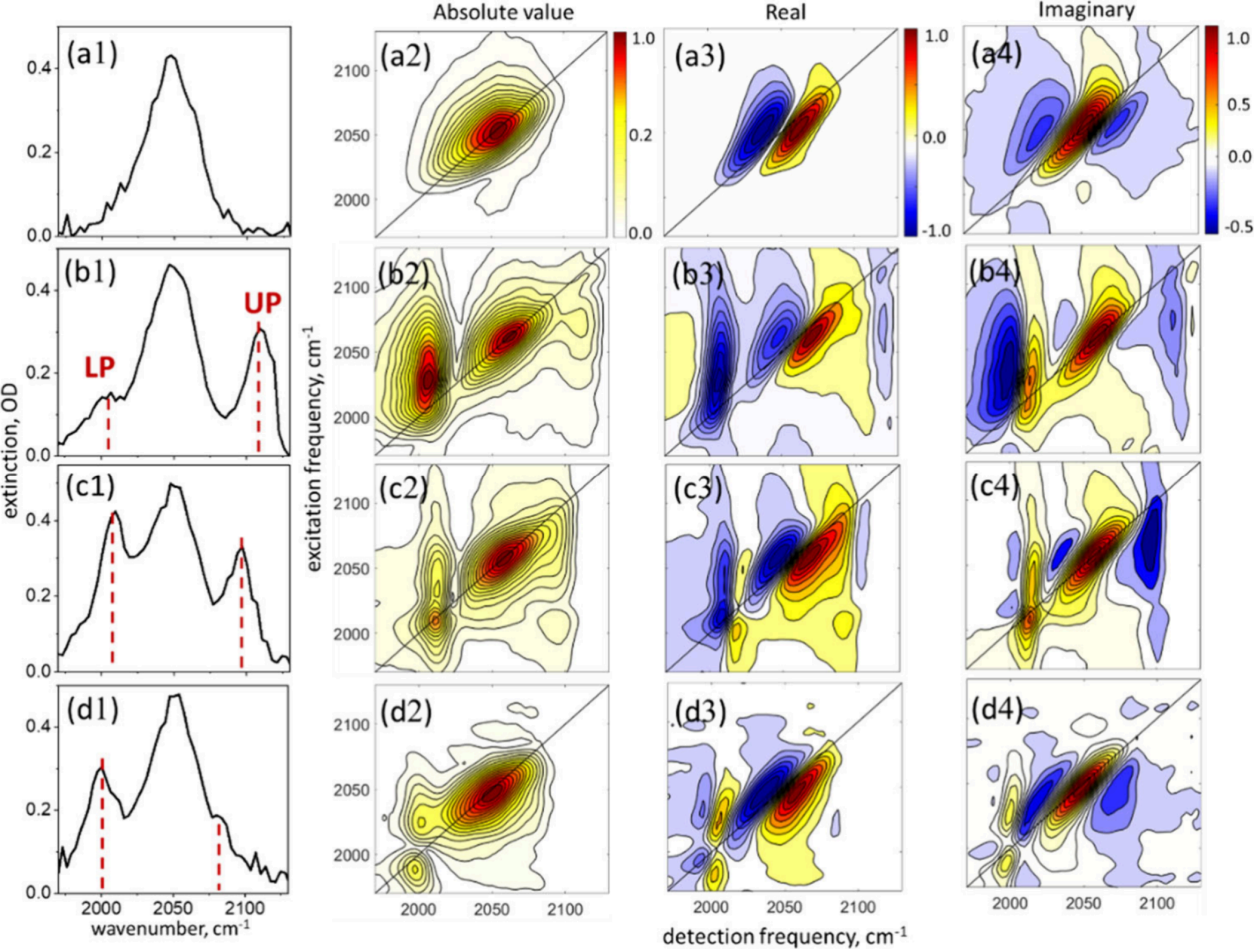
**2DIR spectroscopy of vibro-polaritons.** (a) CN stretching of bare thiocyanate solution. Strongly coupled samples measured with the angles of incidence of the excitation laser pulses θ ≈ 1° (a), 2° (b), and 4° (c), respectively. The left column (panels a1-d1) – the extinction spectrum of the sample obtained from the transmission of the probe laser beam during 2DIR measurements. Panels a2-d2 – the absolute-value 2DIR spectrum. Panels a3–d3 – the real part of the 2DIR spectrum. Panels a4–d4 – the imaginary part of the 2DIR spectrum. The color bars denote normalized amplitudes. All data was collected at the waiting time *T*_w_ = 300 fs.

The characteristic interference pattern of the ground state bleach (GSB) and stimulated emission (SE) transitions at the fundamental frequency with the out-of-phase excited state absorption (ESA) at the overtone frequency, as shown in the real and imaginary parts of the 2DIR spectrum of the CN stretching (Figure 2, panels a3 and a4), is a sensitive observable that allows to determine whether the frequency-selective signal enhancement by the near-fields of vibro-polaritons takes place. Indeed, should such an enhancement mechanism operate solely, no change in phase is expected at the polariton frequencies and the sign of the 2DIR signal would remain similar to that of the bare-molecule sample. Therefore, next, we compare the 2DIR spectra of vibro-polariton samples with those of the bare thiocyanate sample.

The phase-resolved 2DIR spectra of vibro-polariton samples are shown in Figure 2 for cases having different detuning between the SLR and molecular transitions, as controlled by the angle of incidence of the laser pulses. In the left column, the corresponding extinction spectra *S*_ext_ = −log­(*I*/*I*_0_), which were collected during the 2DIR measurements, are also shown for comparison. The case of the near-normal incidence of the laser beams (θ ≈ 1°) is shown in row b of Figure 2, where the Rabi splitting between the coupled states is Ω ≈ 105 cm^–1^. In the absolute-value spectrum in panel b2 we recognize the diagonal UP peak at ω_UP_ = 2110 cm^–1^, the molecular (reservoir) peak around ω_CN_ = 2050 cm^–1^, the diagonal LP peak at ω_LP_ = 2005 cm^–1^, and cross-peaks between the reservoir and each of the polaritons. Whereas in the linear spectrum in panel b1 the UP peak is very strong, in the 2DIR spectrum both the diagonal UP peak and the reservoir-UP cross-peak are rather weak. The phase of the two latter signals is shifted by Δϕ ∼ π with respect to the bare-molecule signal, as seen from inspecting the real and imaginary parts in panels b3 and b4. The LP is much weaker in the linear spectrum, but the corresponding diagonal peak and the reservoir-LP cross-peaks are strong and phase-shifted by Δϕ ∼ π/2. Analysis of the strongly coupled system with the TC-like model reveals that the Hopfield coefficients that quantify the composition of the polariton states are not very different for LP and UP; therefore, they are not likely to be the reason for such a large difference in the strength of the nonlinear signals. The calculated Hopfield coefficients are summarized in Table 1 below.

**Table 1:** Hopfield Coefficients for Vibro-Polaritons in Figure 2

Panels in Figure 2	Ω, cm^–1^	${\left|c_{+1,0}^{LP}\right|}^{2}$	${\left|c_{-1,0}^{LP}\right|}^{2}$	${\left|c_{m}^{LP}\right|}^{2}$	${\left|c_{+1,0}^{UP}\right|}^{2}$	${\left|c_{-1,0}^{UP}\right|}^{2}$	${\left|c_{m}^{UP}\right|}^{2}$
b1 – b4	100	0.2	0.2	0.6	0.3	0.3	0.4
c1 – c4	88	0.11	0.35	0.54	0	0.6	0.4
d1 – d4	80	0.07	0.54	0.39	0	0.44	0.56

Examples of the 2DIR spectra obtained with increased angles of incidence of the excitation laser pulses are shown in Figures 2c (θ ≈ 2°) and 2d (θ ≈ 4°). Here, although the intensity of the UP peak reduces in agreement with the linear dispersion map in Figure 1, the intensity of the LP peak increases and the phase shift becomes smaller, such that the line shape appears more similar to the familiar absorptive ones. In panels c2–c4, we also obtained a LP/UP and the complementary UP/LP cross-peaks, as expected for the strongly coupled system. Hopfield coefficients (see Table 1) obtained for this case indicate that the molecular contribution to the polariton states is similar to that in row b, but that the distribution of the photonic weight among the (+1,0) and (−1,0) SLR states is different. In the example in panels d2–d4, the UP signal’s strength is reduced significantly, in agreement with Figure 1e, where the linear spectra of polaritons are shown with the bare-molecule spectrum subtracted, to better emphasize the changes in polariton transitions.

The phases of the LP and UP polariton states obtained from 2DIR spectroscopy are summarized in Figure 3 for their corresponding transition frequencies. The colormap used for the data points encodes the detuning between the molecular transition frequency, ω_CN_, and that of the SLR mode, ω_SLR_. We found that the phase values follow a simple monotonic trend with functional form of an inverse tangent shown in dashed line in the figure, which predicts limiting values of Δϕ ∼ π and Δϕ ∼ π/2 for the LP and UP, respectively, for the case of the ultimately large Rabi splitting, Ω.

**Figure 3:**
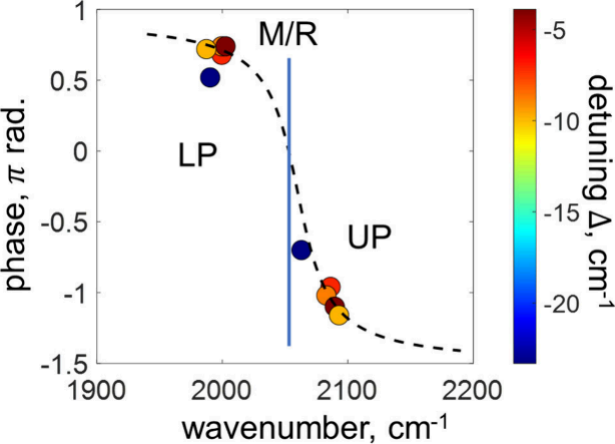
**Phase of polariton signals.** Circles – experimental data obtained with 2DIR spectroscopy. The color of circles encodes the detuning between the molecular transition frequency (vertical line) and that of the SLR mode, Δ = ω_CN_ – ω_SLR_. The data for LP (ω < 2050 cm^–1^) and UP (ω > 2050 cm^–1^) are shown in pairs with matching colors. The trend is indicated as fit to the tan^–1^ function with dashed line.

In order to support the interpretation of the 2DIR spectra of vibro-polaritons as eigenstates of the strongly coupled system, we performed numerical simulations using a standard spectrum-modeling approach based on the nonlinear response functions, which account also for excitation of the coherent superpositions of all the involved states, including the polaritons and reservoir.^48,74^ Figure 4 panels a1–a3 show an example of such a simulated spectrum that qualitatively reproduces all the features in the experimental spectra in rows b and c of Figure 2. In these simulations, polaritons are treated as conventional quantum states with the corresponding transition frequencies ω_LP_ = 2010 cm^–1^, ω_UP_ = 2100 cm^–1^, and homogeneous widths Γ_LP_ = 0.5 ps^–1^ and Γ_UP_ = 1.5 ps^–1^, as estimated from the peak widths of the 2DIR data. For polariton anharmonicities we used the values of their upper bounds of 2 cm^–1^ that were estimated in an earlier work.^49^ Since, as discussed above, the strength of the linear extinction polariton peaks do not necessarily represent their nonlinear strength, polariton transition dipole moments were chosen to qualitatively reproduce the experimental results in Figure 2: μ_CN_ = 1, μ_LP_ = 1, μ_UP_ = 0.5.

**Figure 4:**
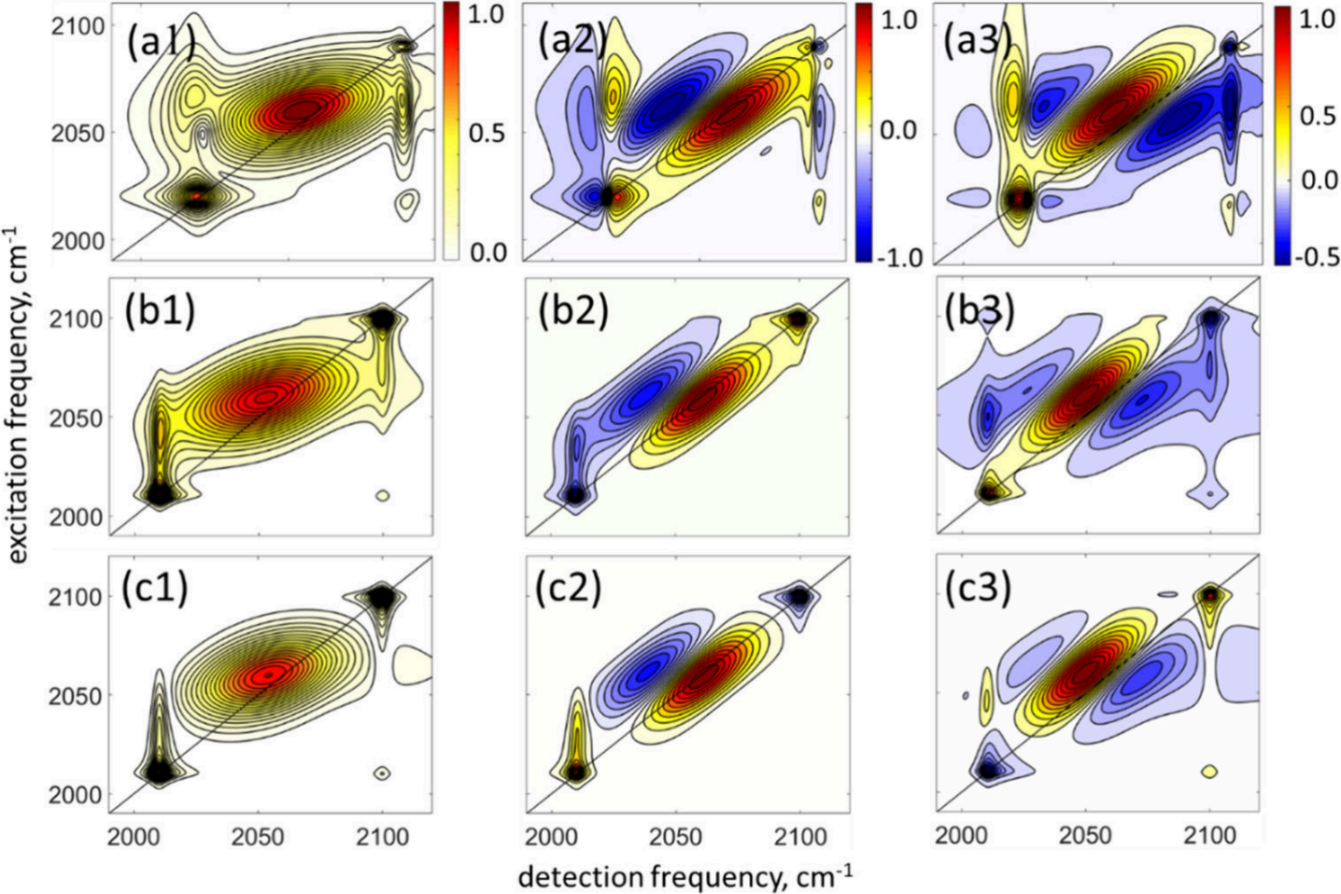
**Simulated 2DIR spectra of vibro-polaritons.** (a) Results obtained with the third-order nonlinear response functions, where polaritons are treated as conventional quantum states and reservoir manifold is considered a bright state. (b) Results obtained with narrowband Lorentzian optical filter functions applied to the bare-molecule spectrum at the polariton frequencies. (c) Results obtained with modeling the spectra in the regime of the Fano-like interference between molecular and polariton transitions. The color bars denote normalized amplitudes.

In contrast, simulations of the bare-molecule spectrum with narrowband enhancements of the signal at polariton frequencies result in qualitatively different 2DIR spectra shown in panels b1–b3 of Figure 4. Assuming that the polariton local field is enhanced 10 times,^69^ the 2DIR signal is expected to be enhanced by 10^4^. The strongest enhancement of the signal occurs on the diagonal line of the 2D spectrum, where polariton transition frequencies are closest to the tails of the inhomogeneously broadened molecular line shape. The cross-peak region is also enhanced, as seen in the figure, especially in the imaginary part of the data, where the dispersive parts of the line shape dominate. However, specific phase relations obtained in the experiment are not reproduced by this approach; therefore, the surface-enhancement mechanism is ruled out as the solely operating one.

We also tested the possibility that molecular transitions weakly couple to polariton resonances and acquire phase twists via the Fano-like interference mechanism.^75^ The Fano phase-twists in the surface-enhanced 2DIR experiments are well-known and can be evaluated quantitatively.^61,62,76^ In our experiments, molecules with low transition strength that would contribute to the enhanced signal via their weak coupling to bright polariton resonances are those whose inhomogeneously distributed vibrational frequencies are resonant with the polaritons. Such resonant condition implies that the corresponding signals would have a phase twist of Δϕ ∼ π at both UP and LP transition frequencies. This scenario is illustrated in Figure 4c; as seen from the figure, the resulted spectra do not agree with the experimental results.

As mentioned above, specific phase relations observed between polariton peaks in the experimental 2DIR spectra (Figures 2b-d, and 3) appear not only because the GSB/SE and the ESA transitions are out-of-phase, but also because polariton transitions acquire phase with respect to the molecular ones. Below, we present the results of the electromagnetic numerical simulations that qualitatively reproduce these phase relations and that further support our conclusions.

## Electromagnetic Simulations

To illustrate the details of polariton waves excitation and propagation, we used the Finite Difference Time Domain method (FDTD-Solution Lumerical, Ansys). A unit cell of the array containing a single gold antenna was used with periodic boundary conditions in the lateral directions. A diffracting planewave source propagating in the direction of normal incidence was used to probe the lattice, and absorbing boundary conditions were used in the transmission and reflection directions. Complex refractive indexes of gold and DMF used were from ref^77^ and ref^71^ respectively, and that of thiocyanate was parametrized from our experimental FTIR measurements. The far-field power distribution versus the relevant in-plane momentum component, *k*_*x*_, was calculated by the Fourier transformation of the field distribution at the near-field monitor; the results are shown in Figure 5 for different excitation frequencies.

**Figure 5:**
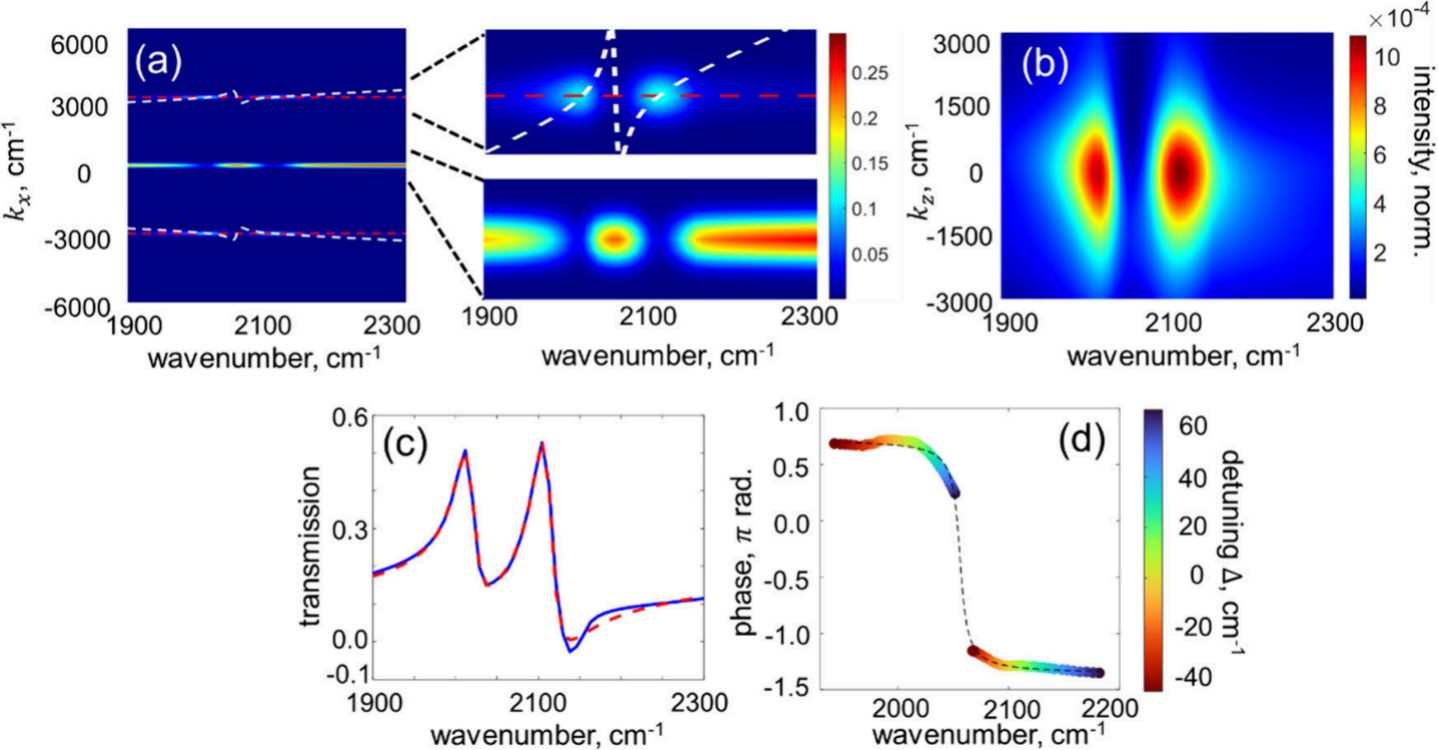
**Electromagnetic numerical simulations of vibro-polaritons.** (a) In-plane momentum dependence of the far-field distribution for different excitation frequencies above the sample surface. The diffraction orders (±1,0) are denoted by red dashed lines to guide the eye. The regions around the zero momentum (transmission) and around the diffraction orders are zoomed-in on the right. The white dashed line denotes the horizon. All data are normalized by the total intensity at the corresponding frequency. (b) Out-of-plane momentum dependence of the far-field distribution calculated for different excitation frequencies in the direction normal to the plane of incidence. (c) Transmission spectrum. Blue solid line – integration of the data in panel a around *k*_*x*_ = 0 with broadband background subtracted. Red dashed line – fit to Lorentzian line shapes with phase. Molecular transition and SLR are in resonance. (d) Phase of polariton signals. The data for LP (ω < 2050 cm^–1^) and UP (ω > 2050 cm^–1^) are shown in pairs with matching colors encoding different detuning Δ = ω_CN_ – ω_SLR_. Dashed line denotes fit to the tan^–1^ function.

As seen in Figure 5a, although most of the power propagates forward (*k*_*x*_ = 0), two distinct diffraction orders (±1,0) are present with momenta matching $\pm n{\mathrm{k⃗}}_{G}$ ; the latter are denoted in red dashed lines to guide the eye. The horizon, which is the line tracing the boundary between the waves propagating in free space and the evanescent waves, given by *k* = *n*(ω)­ω/*c*_0_, is denoted by a white dashed line in the figure, where for *n*(ω) the refractive index of the thiocyanate solution in DMF is used. Two strong peaks appear at the crossing of the diffraction and horizon lines; this indicates that these frequencies are confined to the surface. The resonance condition, where the diffraction order intersects the horizon, is also met at the molecular transition frequency (see also Figure 1c); however, no efficient diffraction is obtained because a strong molecular absorption prevents this mode from propagating across the lattice. The far-field distribution was also calculated in the grazing (x̂) direction; the results are shown in Figure 5b. This projection also exhibits two peaks, with momenta centered at *k*_*z*_ = 0, which indicates again that polaritons propagate along the sample plane. The group velocity in the out-of-plane direction was calculated as *v*_*z*_=∂ω/∂*k*_*z*_. For both polaritons the slope is very small, which further stresses that these modes have no velocity in this direction (ẑ) and are indeed confined to the surface.

Transmission of the strongly coupled sample, which is the quantity measured in the experiments, was calculated by integrating over the nondiffracted portion of the far-field (*k*_*x*_ = 0 in Figure 5a), and normalized to the corresponding transmission without array. The resulting spectrum, shown in Figure 5c with blue solid line, was fitted to a sum of two Lorentzian line shapes with arbitrary phases (red dashed line). Phase-shifts of the molecular transitions on plane metal surfaces and on resonant metallic nanostructures are known both in linear and 2DIR spectroscopy in transmission and reflection configurations.^60–63^ In the present case, the observed phase shifts are associated with the in-plane diffraction and surface-confined nature of the vibro-polaritons; their exact value depends on the detuning between the SLR and molecular transition frequencies. To illustrate this effect, the phases of the polariton peaks calculated with FDTD are shown in Figure 5d for different detuning Δ*b*etween the molecular and the SLR transitions. The corresponding pairs of the phase values are denoted with circles of the matching colors, which encode the magnitude of the detuning. The obtained phase shifts follow a functional form of the inverse tangent, as shown with the dashed line in the figure, similar to experimental results in Figure 3.

The results of the phase-resolved 2DIR spectroscopy measurements presented above indicate that nonlinear signals from vibro-polaritons, formed when molecular vibrations strongly couple to the SLR photonic modes of the infrared antenna array, represent the associated quantum states of the many-body system. While linear phase-resolved spectroscopy was recently shown to allow for the phase topology analysis to reveal strong coupling in the congested spectra,^78–80^ the phase-resolved 2DIR spectra demonstrated in this work not only provide the complex-valued response, but also reveal the cross-peaks between the quantum states reflecting their coupling and correlation. As such, we demonstrated that the excitation of the reservoir manifold, which is frequently treated as bare molecular, is correlated with both polariton states, emphasizing that these states belong to the same strongly coupled system.

Our observations can be compared to experiments with van der Waals semiconductor meta-surfaces, where reservoir-polariton cross-peaks were recently detected with 2D spectroscopy in the visible spectral region and attributed to the presence of two distinct exciton types.^81^ Herein, we interpret our results within the framework where the interactions between vibro-polaritons and between polaritons and reservoir states occur via the corresponding anharmonic constants and achieve qualitative agreement with a model based on the third-order nonlinear response functions, which treats the polaritons as proper quantum states of the strongly coupled system. That is, we found that for effective anharmonicities of 2 cm^–1^, this model reproduces the phase-resolved 2DIR line shapes obtained in our experiments, whereas models involving enhancement of molecular signals by polariton fields and weak coupling to polariton resonances disagree with the experimental data.

We note that in contrast to the ingredients of typical Tavis-Cummings-like models of higher excitation tiers,^47^ molecular anharmonicity is not the only source of the nonlinearity in the system discussed in the present work: both metallic nanostructures and their periodic arrays are well-known to produce nonlinear signals, including second and third harmonics^82,83^ The third-order nonlinear susceptibility reported for resonant gold nanostructures^84^ reaches χ^(3)^∼10^–17^ m^2^/V^2^, which is on par with molecular nonlinearity in our samples.^48^ If molecular anharmonicity contribution to the collective state, which is spread over many molecules, would vanish^47^ analogous to the reduction of anharmonicity with the participation number in vibrational excitons in biomolecules,^85^ the gold antennas would solely provide polariton’s nonlinearity observed in our experiments. These considerations apparently contradict our observations based on comparison between the experimental results and the response functions model; however, they suggest that any model aiming to describe nonlinear response in SLR-based vibro-polaritons should account also for the nonlinear response of the photonic resonator.

The distinct quantum state nature of molecular polaritons together with the surface-bound character of the polariton waves, can be potentially related to the actively debated processes of polaritonic chemistry activated by fluctuating vacuum fields. A momentum-space description that we invoked provides a convenient framework for analyzing polaritons confined to meta-surfaces; the details of their propagation and relaxation will be investigated further by nonlinear microspectroscopy.

## Supplementary Material



## Data Availability

All data needed to evaluate the conclusions in the paper are present in the paper.

## Notes

The Tavis-Cumming-like model of linear vibro-polariton spectra. Polariton sample preparation and characterization. 2DIR spectroscopy (PDF)
