# Supplementary material for: Phase-Resolved Two-Dimensional Infrared Spectroscopy of Solution-Phase Vibrational Polaritons on Gold Antenna Meta-Surfaces
Source: J Phys Chem Lett. 2026 Feb 22;17(9):2644–53. doi: 10.1021/acs.jpclett.6c00009 (PMC12969361; doi:10.1021/acs.jpclett.6c00009)
Supplement: Supplementary file 1 [file jz6c00009_si_001.pdf]

Phase-resolved two-dimensional infrared spectroscopy of solution-phase vibrational polaritons on gold antenna meta-surfaces

*Shmuel Sufrin, Bar Cohn, and Lev Chuntonov**

Schulich Faculty of Chemistry, Technion - Israel Institute of Technology, Haifa 3200003, Israel; Solid State Institute, Technion - Israel Institute of Technology, Haifa 3200003, Israel; The Helen Diller Quantum Center, Technion - Israel Institute of Technology, Haifa 3200003, Israel

email: chunt@technion.ac.il

Supplementary Information

1. The Tavis-Cumming-like model of linear vibro-polariton spectra

The Hamiltonian describing the interaction between the *N* molecular vibrational transitions and the SLR modes used in the present work is given by

$$\hat{H}_0 = \hat{H}_{SLR} + \hat{H}_{mol} + \hat{H}_{int},$$

where $\hat{H}_{SLR}$ describes the SLR modes, $\hat{H}_m$ describes the molecules, and $\hat{H}_I$ describes the interaction between the two parts. The SLR Hamiltonian is given by

$$\hat{H}_{SLR} = \hbar\omega_{0,+1}(k_\parallel)c^\dagger_{0,+1}c_{0,+1} + \hbar\omega_{0,-1}(k_\parallel)c^\dagger_{0,-1}c_{0,-1} + g_{SLR}\left[c^\dagger_{0,+1}c_{0,-1} + c^\dagger_{0,-1}c_{0,+1}\right],$$

where the SLR modes are indexed by their diffraction order components (0,+1) and (0,-1), $\omega_{SLR}(k_\parallel)$ is the SLR transition frequency, $k_\parallel$ is the in-plane momentum, $c_{SLR}(c^\dagger_{SLR})$ are the corresponding ladder operators, and g_{SLR} is the coupling between the (0,+1) and the (0,-1) modes. The molecular Hamiltonian is given by

$$\hat{H}_{mol} = \sum_{i=1}^{N} \hbar\omega_i^m\, b_i^\dagger b_i\,,$$

where ω_i^m is the fundamental transition frequency of the *i*-th molecule within the inhomogeneous ensemble of *N* molecules, and $b_i(b_i^\dagger)$ are the corresponding ladder operators.

The interaction term is given by

$$\hat{H}_{int} = \sum_{v=1}^{V}\sum_{l=1}^{L}\sum_{m=1}^{M} g^{0,+1}_{v,l,m}\left(c^\dagger_{0,+1}b_{v,l,m} + b^\dagger_{v,l,m}c_{0,+1}\right) + g^{0,-1}_{v,l,m}\left(c^\dagger_{0,-1}b_{v,l,m} + b^\dagger_{v,l,m}c_{0,-1}\right),$$

where the coupling constants g_i^j describe the strength of the interaction between the molecule *i* and the SLR mode *j*. The coupling constants are given by $g_i^j = \vec{\mu}_i \cdot \vec{E}_{loc}(\vec{r}, \omega_j)$, where $\vec{\mu}_i$ is the transition dipole moment of the molecule and $\vec{E}_{loc}(\vec{r}, \omega_j)$ is the electric field at the SLR frequency ω_j at the molecular location $\vec{r}$, which was obtained with electromagnetic numerical simulations. The representative distribution of the near field intensity is shown in Figure S1 below, along with the distribution of the corresponding strength of the coupling constants. Summation over the molecules is divided into several parts: First, the index *v* enumerates the volume elements (voxels), where the field $\vec{E}_{loc}(\vec{r}_v)$ was evaluated, then, the index *m* samples the isotropic orientation of molecular transition dipole moments within each voxel. Finally, inhomogeneous distribution of vibrational frequencies ω_m, which follows $e^{-\frac{(\omega_m - \omega_0)^2}{2\sigma^2}}$, is sampled by index m. Overall, $V \times L \times M = N$, where *N* is the total number of molecules.

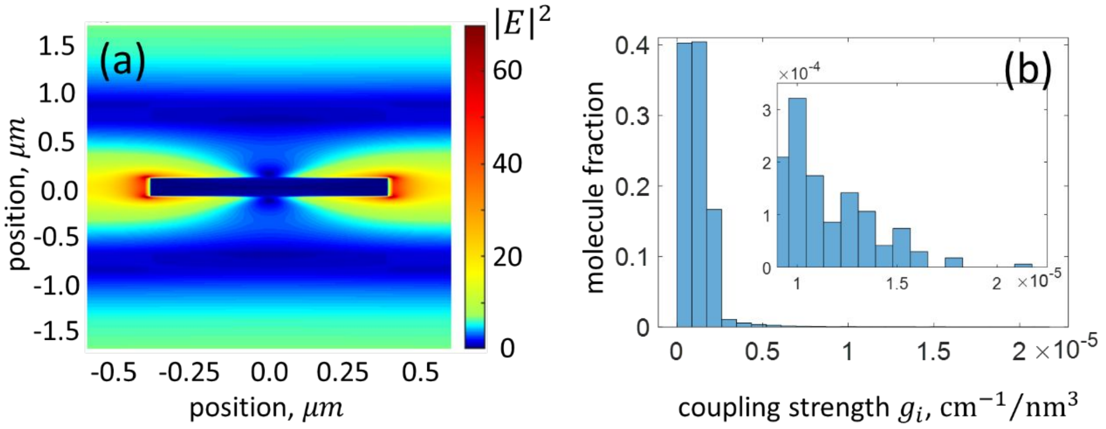

Figure S1: (a) Map of the near-field distribution for SLR at the normal incidence. (b) Distribution of the coupling strength constants. The values are normalized to the unit of volume.

2. Materials and Methods

Polariton sample preparation and characterization

Arrays of half-wavelength gold micro-antennas were fabricated by electron beam lithography on a 2 mm-thick CaF_2 substrate (Crystran). The antenna dimensions were *L*=750

nm (length), W=200 nm (width), and H=80 nm (height); the lattice period was Λ_x=3.4 μm and Λ_y =1.2 μm, as imaged with scanning electron microscopy (see Figure 1a).

FTIR spectroscopy (Nicolet iS10, Thermo-Fisher) of the antenna array was performed with the sample covered by a ca. 500 nm-thick film of dimethyl formamide (DMF) solvent and capped with another CaF_2 window; the measured quality factor of the SLR was Q=150 at the normal incidence, as shown in Figure 1b. Polariton samples were prepared with a 2M solution of ammonium thiocyanate in *N,N*-dimethyl formamide solvent (DMF) and measured with light polarized along the antenna axis. The angle between the excitation beam and the array was controlled with a pair of goniometers. A pair of ca. 2 mm apertures was set before and after the sample to narrow the distribution of the angles of incidence naturally present in the beam of the FTIR spectrometer.

2DIR spectroscopy

The polaritons' dispersion relations indicate a strong dependence of their transition frequencies on the angle of incidence of the TE-polarized excitation light, as illustrated in Figure 1. In agreement with earlier results,[1] the sample has a very weak dispersion with the polar angle (TM polarization), which can be neglected for small angles used for the excitation laser beams in our experiments. The dispersion is strong with the azimuthal angle, as seen in Figure 1. For 2DIR measurements, each of the three laser beams must achieve identical excitation conditions simultaneously. The two-beam configuration of the 2DIR optical setup simplifies measurements compared with the box-like four-wave mixing configuration.[2,3]

Mid-infrared laser pulses with a time duration of ca. 70 fs (Solstice, Spectra Physics; TOPAS, Light conversion, rep. rate 4KHz) were split into two replicas and recombined colinearly with beam splitters in a Michelson-type interferometer. The time interval between these pulses was systematically scanned with a translational delay stage and used to generate the excitation frequency axis of the 2D spectrum via the corresponding Fourier transformation. The excitation pulses were chopped and the differential transmission of the third weak probe pulse was recorded with a spectrograph equipped with an infrared camera (Phasetech). Additional phase cycling was performed with a pair of wobblers to

eliminate scattered light.[4] We verified that both the pump and probe beams have identical transmission spectra in each measurement.

In 2DIR instruments using an acousto-optic modulator to generate the excitation pulse pair,[5] the time interval between the pulses is controlled to a very high precision and the phase of the 2DIR signal is locked. However, when mechanical delay stages are used, the signal phase, which is needed to obtain the desired absorptive line shapes, is found with the help of a reference signal, e.g., a transient absorption spectrum.[6] When the latter is not available in cases of very weak signals or highly scattering samples, 2DIR spectra are phased with an internal reference – a signal with a well-known 2D line shape present in the sample. This is the approach used in the present work: Since static parasitic light scattered by the probe laser beam could not be removed completely, the signal's phase was referenced to the bare-molecule (reservoir) transitions that were naturally present in all measured data.

References

(1) Cohn, B.; Das, K.; Basu, A.; Chuntonov, L. Infrared Open Cavities for Strong Vibrational Coupling. *J. Phys. Chem. Lett.* **2021,** *12*, 7060.

(2) Cohn, B.; Sufrin, S.; Chuntonov, L. Ultrafast vibrational excitation transfer on resonant antenna lattices revealed by two-dimensional infrared spectroscopy. *J. Chem. Phys.* **2022,** *156* (12), 121101.

(3) Sufrin, S.; Cohn, B.; Chuntonov, L. Probing the anharmonicity of vibrational polaritons with double-quantum two-dimensional infrared spectroscopy. *Nanophotonics* **2024,** *13* (14), 2523.

(4) Bloem, R.; Garrett-Roe, S.; Strzalka, H.; Hamm, P.; Donaldson, P. Enhancing signal detection and completely eliminating scattering using quasi-phase-cycling in 2D IR experiments. *Opt. Express* **2010,** *18* (26), 27067.

(5) Shim, S.-H.; Strasfeld, D. B.; Ling, Y. L.; Zanni, M. T. Automated 2D IR spectroscopy using a mid-IR pulse shaper and application of this technology to the human islet amyloid polypeptide. *Proc. Nat. Acad. Sci.* **2007,** *104* (36), 14197.

(6) Hamm, P.; Zanni, M. T. *Concepts and Methods of 2D Infrared Spectroscopy*; Cambridge University Press: Cambridge, 2011.
